# Synthetic Ruthenium Complex TQ-6 Potently Recovers Cerebral Ischemic Stroke: Attenuation of Microglia and Platelet Activation

**DOI:** 10.3390/jcm9040996

**Published:** 2020-04-02

**Authors:** Chih-Hsuan Hsia, Thanasekaran Jayakumar, Joen-Rong Sheu, Chih-Wei Hsia, Wei-Chieh Huang, Marappan Velusamy, Li-Ming Lien

**Affiliations:** 1 Translational Medicine Center, Shin Kong Wu Ho-Su Memorial Hospital, Taipei 111, Taiwan; T014913@ms.skh.org.tw; 2 Graduate Institute of Medical Sciences and Department of Pharmacology, College of Medicine, Taipei Medical University, Taipei 110, Taiwan; tjaya_2002@yahoo.co.in (T.J.); sheujr@tmu.edu.tw (J.-R.S.); d119106003@tmu.edu.tw (C.-W.H.); m120107013@tmu.edu.tw (W.-C.H.); 3 Department of Chemistry, North Eastern Hill University, Shillong 793022, India; mvelusamy@gmail.com; 4 Department of Neurology, School of Medicine, College of Medicine, Taipei Medical University, Taipei 110, Taiwan; 5 Department of Neurology, Shin Kong Wu Ho-Su Memorial Hospital, Taipei 111, Taiwan

**Keywords:** BV2 microglia, brain infarct/edema, neuroinflammation, Nrf2/HO-1, NF-κB, platelets, ruthenium complex

## Abstract

Activated microglia are crucial in the regulation of neuronal homeostasis and neuroinflammation. They also contribute to neuropathological processes after ischemic stroke. Thus, finding new approaches for reducing neuroinflammation has gained considerable attention. The metal ruthenium has gained notable attention because of its ability to form new complexes that can be used in disease treatment. [Ru(η6-cymene)2-(1H-benzoimidazol-2-yl)-quinoline Cl]BF4 (TQ-6), a potent ruthenium (II)-derived compound, was used in this study to investigate its neuroprotective action against microglia activation, middle cerebral artery occlusion (MCAO)-induced embolic stroke, and platelet activation, respectively. TQ-6 (2 μM) potently diminished inflammatory mediators (nitric oxide/inducible nitric oxide synthase (iNOS) and cyclooxygenase 2 (COX-2)) expression, nuclear factor kappa B (NF-κB) p65 phosphorylation, nuclear translocation, and hydroxyl radical (OH^•^) formation in LPS-stimulated microglia. Conversely, TQ-6 increased the expression of nuclear factor erythroid 2-related factor 2 (Nrf2) and heme oxygenase-1 (HO-1). Moreover, it significantly reduced brain infarct volume and edema in MCAO mice. Additionally, it drastically inhibited platelet aggregation and OH^•^ production in mice platelets. This study confirmed that TQ-6 exerts an anti-neuroinflammatory effect on microglia activation through neuroprotection, antiplatelet activation, and free radical scavenging. The authors propose that TQ-6 might mitigate neurodegenerative pathology by inhibiting the NF-κB-mediated downstream pathway (iNOS and COX-2) and enhancing Nrf2/HO-1 signaling molecules in microglia.

## 1. Introduction

Microglia are the chief macrophages associated with inflammatory reaction in the central nervous system (CNS), and are essential regulators of homeostasis and neuroinflammation [1]. In abnormal states, such as infection or inflammation, microglia are triggered, leading to the discharge of various inflammatory mediators, such as reactive oxygen species (ROS) and nitric oxide (NO) [2]. Consequently, microglia hyperactivities are connected to the pathologies of several CNS diseases, including ischemic stroke; microglia perform different physiological roles when stimulated [3]. M1 microglia activation causes tissue damage, resulting in chronic neurodegeneration, whereas M2 microglia activation diminishes inflammation, leading to tissue repair and regeneration [3,4]. Inflammation occurs during ischemic stroke and plays a vital role in neuropathological disorders [5]. After an ischemic stroke, microglia stop other processes and secrete inflammatory cytokines [6]. LPS induces the expression of M1 markers, such as cyclooxygenase-2 (COX-2), inducible nitric oxide synthase (iNOS), reactive oxygen species (ROS) such as nitric oxide (NO), and several proinflammatory cytokines including interleukin (IL)-1β [7]. By contrast, arginase 1 and CD206 (a mannose receptor), which are markers of M2 that localize in the inflammatory zone, block the expression of proinflammatory mediators such as iNOS, and promote neuroprotection [8].

Recent studies have suggested that stimulating intracellular anti-inflammatory mechanisms, such as nuclear factor erythroid 2-related factor 2 (Nrf2) signaling, could inhibit inflammation-mediated neuronal toxicity. Neuroinflammatory M1 markers COX-2 and iNOS increased and M2 markers (IL-4, IL-10, and Arg1) decreased in response to neurotoxins in Nrf2-deficient mice [9,10]. Moreover, other investigations have suggested that nuclear factor kappa B (NF-κB) is a principal regulator of the M1 phenotype, whereas Nrf2 may be a regulator of the M2 phenotype [10]. Some studies have identified Nrf2 as being relevant in microglia immunomodulation; particularly, heme oxygenase-1 (HO-1) is crucial for cell protection in response to oxidative stress during neuroinflammation [11]. HO-1 activation suppresses lipopolysaccharide (LPS)-induced inflammation in mouse peritoneal macrophages and microglia [12]; microglial activation was observed in the penumbra after middle cerebral artery occlusion (MCAO) injury [13]. In recent decades, many investigators have attempted to improve pathology after stroke by normalizing microglia function and altering the microglia phenotype during inflammation by using various substances [14]. Thus, targeting Nrf2/HO-1 with appropriate pharmacological compounds may diminish neuroinflammation and related neurodegeneration.

Organometallic ruthenium complexes are beneficial against various types of cancer, and are frequently considered potential substitutes for current therapeutic approaches [15]. Maysinger et al. [16] reported on the cytotoxicity of a series of ruthenium complexes. Ruthenium-containing complexes such as NAMI-A, KP1019, and KP1339 have been used in clinical trials, whereas the complex DW1/2 has not advanced past the preclinical phase [17]. A considerable number of ruthenium metal compounds have been identified as effective antiplatelet agents for preventing and treating thrombotic diseases [18,19]. Thus, metal complexes are potential alternatives to anti-inflammatory drugs. This study investigated the capacity of a novel ruthenium complex [Ru(η6-cymene)2-(1H-benzoimidazol-2-yl)-quinoline Cl]BF4 (TQ-6) to prevent LPS-induced inflammation in microglia, MCAO-induced ischemic stroke, and platelet activation in mice; additionally, it elucidated the underlying defensive mechanisms of TQ-6 by examining the participation of NF-κB and Nrf2/HO-1 signaling molecules in these experiments.

## 2. Materials and Methods

### 2.1. Materials

Dulbecco’s modified Eagle medium (DMEM), fetal bovine serum (FBS), L-glutamine/penicillin/streptomycin, and anti-α-tubulin monoclonal antibodies (mAbs) were purchased from Invitrogen (Thermo Fisher Scientific, Waltham, MA, USA). LPS and 3-(4,5-dimethylthiazol-2-yl)-2,5-diphenyltetrazolium bromide (MTT) were bought from Sigma-Aldrich (St. Louis, MO, USA). Anti-iNOS, COX2, and Nrf2 polyclonal antibodies (pAbs) were purchased from Santa Cruz Biotechnology (Dallas, TX, USA). An anti-HO-1 pAb was purchased from Enzo (Farmingdale, New York, NY, USA). The anti-phospho-p65 (Ser536) mAb was purchased from Cell Signaling (Danvers, MA, USA). Horseradish peroxidase (HRP)-conjugated donkey anti-rabbit immunoglobulin G (IgG) and sheep anti-mouse IgG were purchased from Amersham (Buckinghamshire, U.K.). Enhanced chemiluminescence (ECL) Western blotting detection reagent and Hybond-P polyvinylidene difluoride (PVDF) blotting membranes were purchased from GE Healthcare Life Sciences (Waukesha, WI, USA).

### 2.2. TQ-6 Synthesis and Microglia Cultivation

**Figure 1:**
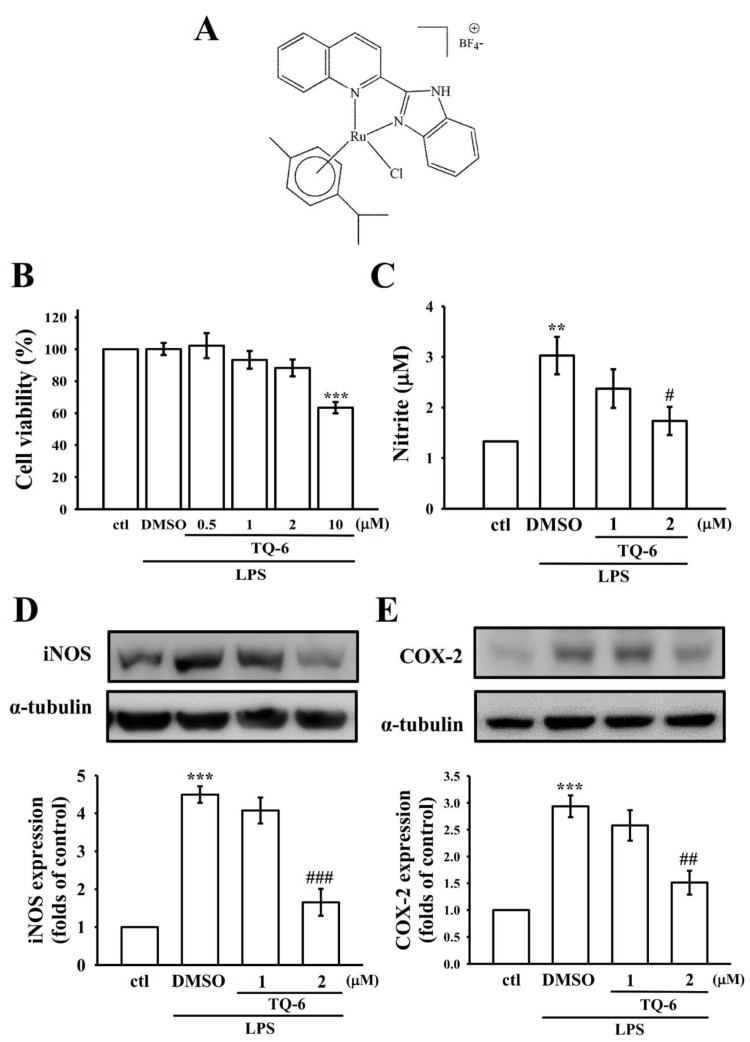
Effects of [Ru(η6-cymene)2-(1H-benzoimidazol-2-yl)-quinoline Cl]BF4 (TQ-6) on cell viability, nitric oxide (NO) production, inducible nitric oxide synthase (iNOS), and cyclooxygenase 2 (COX-2) expression in lipopolysaccharide (LPS)-stimulated microglia. (**A**) Chemical structure of TQ-6. Cells were pretreated with TQ-6 (0.5–10 μM) or 0.1% dimethyl sulfoxide (DMSO) for 30 min and then treated with LPS (1 μg/mL) for 24 h to measure (**B**) cell viability using a 3-(4,5-dimethylthiazol-2-yl)-2,5-diphenyltetrazolium bromide (MTT) assay, (**C**) NO (nitrite) by using a Griess reagent, and (**D**) iNOS or (**E**) COX-2 protein expression by using an immunoblotting assay. Data are presented as mean ± standard error of the mean (SEM; *n* = 4); ** *p* < 0.01 and *** *p* < 0.001 compared with control (ctl); ^#^ *p* < 0.05, ^##^ *p* < 0.01, and ^###^ *p* < 0.001 compared with the LPS-treated cells.

TQ-6 (Figure 1A) and its ligand (L) were synthesized according to the method used in a previous study [18]. The BV2 microglia were gifted by Professor Lin, Department of Pharmacology, School of Medicine, National Taiwan University. The cells were cultured in DMEM supplemented with 10% FBS, 100 U/mL penicillin G, and 100 mg/mL streptomycin at 37 °C in a humidified atmosphere of 5% CO_2_/95% air [20].

### 2.3. Cell Viability Assay

Microglia were plated into 24-well culture plates at 1 × 10^5^ cells/well and cultured in DMEM. After incubating for one day, the cells were treated with either the solvent control (0.1% dimethyl sulfoxide, DMSO) or TQ-6 (1 or 2 µM) for 30 min, and then stimulated using LPS (1 µg/mL) for 24 h. Cell viability was measured using an MTT assay [18], and the viability index was calculated as follows: (absorbance of treated cells/absorbance of control cells) × 100%. The absorbance of the samples was determined at 570 nm by using an MRX absorbance reader (Dynex Technologies, Chantilly, VA, USA).

### 2.4. Determination of NO Production

The NO production was measured using a previously described method with slight alterations [20]. A total of 8 × 10^5^ microglia were seeded in 6-cm dishes with DMEM for 24 h. The cells were treated with TQ-6 (1 or 2 μM) or 0.1% DMSO for 30 min, with or without LPS (1 μg/mL) for 24 h. The supernatants were collected and mixed with equal volumes of Griess reagent. The absorbance of the samples was determined at 550 nm. The NO concentration was calculated using a standard curve obtained through the linear regression of absorbance measurements of standard solutions.

### 2.5. Western Blotting Analysis

Western blotting analysis was performed as previously described [20]. Microglia (8 × 10^5^ cells/dish) were seeded in 6-cm dishes with DMEM for 24 h, and the cells were pretreated with TQ-6 for 30 min, with or without LPS (1 μg/mL). Further, cellular proteins were extracted using a lysis buffer. In total, 50 μg of proteins were subjected to sodium dodecyl sulfate-polyacrylamide gel electrophoresis, and the separated proteins were transferred onto 0.45-μm PVDF membranes. Skimmed milk (5%) and Tris-buffered saline with Tween 20 buffer were added to block the membranes for 30 min, which were then probed with the primary antibodies for 2 h and exposed to HRP-conjugated sheep anti-mouse IgG for 1 h. The immune-reactive bands were detected using the ECL system. The density of protein bands was measured using the Biolight Windows Application V2000.01 (Bio-Profil, Vilber Lourmat, France).

### 2.6. Confocal Immunofluorescence Staining Assay

Microglia (1 × 10^5^ cells/well) were cultured on cover slips in six-well plates and treated with 0.1% DMSO or 2 μM TQ-6 in the presence or absence of LPS for 30 min. The cells were washed with phosphate buffered saline (PBS) and fixed with 4% paraformaldehyde for 10 min. After incubation, the cells were permeabilized with 0.1% Triton X-100 for 10 min and blocked with 5% bovine serum albumin for 30 min. Subsequently, the cells were incubated with an anti-NF-κB p65 mAb for 2 h, washed with PBS, and then subjected to fluorescein isothiocyanate (FITC)-conjugated anti-rabbit IgG for 1 h. They were stained with 4’,6-diamidino-2-phenylindole (DAPI; 30 μM) and mounted on a glass slide using a mounting buffer (Vector Laboratories). The fluorescence images were captured using a Leica TCS SP5 Confocal Spectral Microscope Imaging System (Mannheim, Germany) [21].

### 2.7. Animals

All C57BL/6 mice (male, 25–30 g) were purchased from BioLASCO (Taipei, Taiwan). All of the animal experiments and procedures were performed according to the provisions of the Institutional Animal Care and Use Committee of Taipei Medical University (approval no. LAC-2018-0360). The mice were maintained under controlled environmental conditions of 24 ± 1 °C ambient temperature, 55% ± 10% relative humidity, and a 12-h light/dark cycle, and had free access to food and water. All animals were clinically confirmed to be normal and free from apparent infection, inflammation, or neurological deficits.

### 2.8. Middle Cerebral Artery Occlusion-Induced Cerebral Ischemia in Mice

The mice were anesthetized with 75% air and 3% isoflurane, and then maintained in a 25% oxygen environment. The rectal temperature was maintained at 37 ± 0.5 °C. The mice were subjected to transient focal cerebral ischemia, which was instigated by blocking their right middle cerebral artery (MCA), as described in a previous study [21]. The right common carotid artery was exposed, and a 6-0 monofilament nylon thread (20 mm) coated with silicon (3 mm) was inserted from the external to the internal carotid artery, until the tip occluded the MCA origin. After closing the site of the operation, anesthesia was terminated, and the mice regained consciousness. During another brief period of anesthesia, the filament was gently removed after a 30-min middle cerebral artery occlusion (MCAO). The mice were divided into four groups, which received the following treatments: (1) a sham operation, (2) intraperitoneal DMSO followed by MCAO, and (3 and 4) administration of TQ-6 (50 and 100 μg/kg, respectively) followed by MCAO. All of the treatments were administered before MCAO, except in the sham operation group.

### 2.9. Measurement of Brain Infarct Volume and Edema

The brains of the treated mice were directly removed, rinsed in cold saline solution, and sliced into coronal sections of 2-mm thick. The slices were immediately immersed in 2% 2,3,5-triphenyltetrazolium chloride (TTC) for 30 min, followed by an overnight fixation in 4% formaldehyde. The TTC-stained sections with the viable cerebral tissue stained red and the infarcted cerebral tissue remaining pale were photographed; the infarcted areas of each section were measured using the Image Proplus (version 6.0; Media Cybernetics, Rockville, MD, USA) analyzer. To compensate for edema formation in the ipsilateral hemisphere, infarct volumes were expressed as a percentage of the contralateral hemisphere volume, calculated as follows: infarct volume = (area of the intact contralateral left hemisphere) − (area of the intact ipsilateral right hemisphere) [21]. The edema ratio was calculated as follows: edema ratio = (ischemic volume − nonischemic volume)/(ischemic volume + nonischemic volume) × 100% [21]. No mortality occurred during the experimental periods of the selected dose administration.

### 2.10. Platelet Aggregation

Platelet suspensions were prepared according to the methods used in a previous study [22]. The blood obtained from male C57BL/6 mice was mixed with a 3.8% sodium citrate solution. After centrifugation, the supernatant enriched with platelet-rich plasma (PRP) was measured using a lumi-aggregometer (Payton Associates, Scarborough, ON, Canada) [23]. After centrifugation of the PRP, the platelet suspensions were examined for free radicals.

### 2.11. Detection of Hydroxyl Radicals through Electron Spin Resonance Spectrometry

According to the method described in an earlier study [24], electron spin resonance (ESR) spectrometry was performed using a Bruker EMX ESR spectrometer (Billerica, MA, USA). Both microglia (5 × 10^5^ cells/Eppendorf tube) and mice platelet suspensions (2 × 10^8^ cells/mL) were pretreated with 0.1% DMSO or TQ-6 (1 or 2 μM) for 6 min, followed by treatment with LPS (1 μg/mL) and collagen (2 μg/mL), respectively. The ESR spectrometer was operated at a power of 20 mW, a frequency of 9.78 GHz, a scan range of 100 G, and a receiver gain of 5 × 10^4^; then, 100 μM 5,5-dimethyl-1-pyrroline-N-oxide was added before conducting the ESR analysis.

### 2.12. Statistical Analysis

The results are expressed as mean ± standard error of the mean (SEM), along with the number of observations (*n*), where *n* refers to the number of experiments. An unpaired Student’s *t*-test or analysis of variance was used to determine significant differences between the groups, and the groups with significant differences were compared using the Student–Newman–Keuls method. Statistical significance was set at *p <* 0.05.

## 3. Results

### 3.1. TQ-6 Reduced NO Production and Attenuated Inflammatory Markers in Microglia

To evaluate the cellular toxicity of TQ-6, cell viability was measured in microglia treated with TQ-6 using an MTT assay. The results revealed that TQ-6 (1 or 2 µM) did not affect the viability of the microglia (Figure 1B), and hence, TQ-6 was used at concentrations of 1 or 2 µM in further studies. Moreover, this study revealed that NO production was increased in microglia after LPS treatment; however, 2 µM TQ-6 significantly reduced this elevation (*p* < 0.05; Figure 1C). To assess the stimulation of iNOS and COX-2 inflammatory mediators in LPS-treated microglia, Western blotting analysis was performed. In LPS-treated cells, the iNOS and COX-2 levels increased significantly; however, TQ-6 pretreatment significantly (*p* < 0.01) inhibited the expression of both these proteins (Figure 1D,E).

### 3.2. NF-κB p65 Phosphorylation and Nuclear Translocation Were Suppressed by TQ-6

**Figure 2:**
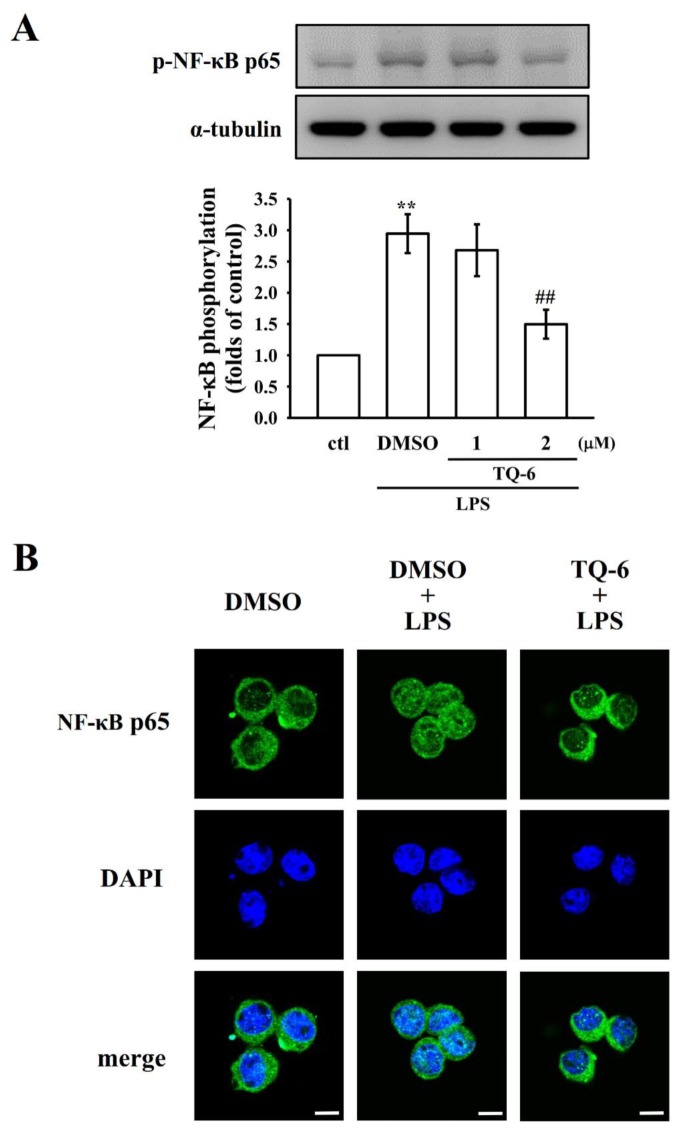
Inhibitory effect of TQ-6 on NF-κB p65 phosphorylation and nuclear translocation in LPS-stimulated microglia. (**A**) Cells were treated with 0.1% DMSO or TQ-6 (1 or 2 μM), followed by LPS (1 μg/mL) for 30 min for immunoblotting assay. (**B**) The immunofluorescence analysis was performed with an anti-NF-κB p65 antibody and fluorescein isothiocyanate (FITC)-conjugated anti-rabbit immunoglobulin G (IgG; green). 4’,6-diamidino-2-phenylindole (DAPI) was used to label the nuclei (blue). The images were captured through confocal microscopy (scale bar = 7.5 μm). Data are presented as mean ± SEM (*n* = 4). ** *p* < 0.01 compared with control (ctl); ^##^ *p* < 0.01 compared with the LPS-treated cells.

NF-κB is a key regulator of inflammation, platelet aggregation, and atherogenesis [25]. The activation of NF-κB signaling is closely associated with an increase in the levels of proinflammatory proteins such as iNOS and COX-2. Because TQ-6 potently inhibits these proteins, this study examined the effect of TQ-6 on phosphorylated NF-κB p65 expression and nuclear translocation in LPS-treated microglia. TQ-6 treatment considerably reduced the phosphorylation of NF-κB p65, as presented in Figure 2A. Next, a confocal immunofluorescence assay was carried out to confirm the inhibition of LPS-induced p65 nuclear translocation by TQ-6 in microglia. Compared with the control cells, LPS treatment markedly increased p65 nuclear export, as evidenced by the amplified FITC labeled NF-κB p65 in the cell nuclei (green fluorescence). DAPI was used to label the nuclei (blue fluorescence). However, treatment with TQ-6 at 2 μM significantly blocked the nuclear translocation of p65, which was confirmed by noticing reduced green fluorescence in the nuclear fraction.

### 3.3. TQ-6 Enhanced LPS-Mediated Nrf2/HO-1 Expression and Inhibited OH^•^ Formation

**Figure 3:**
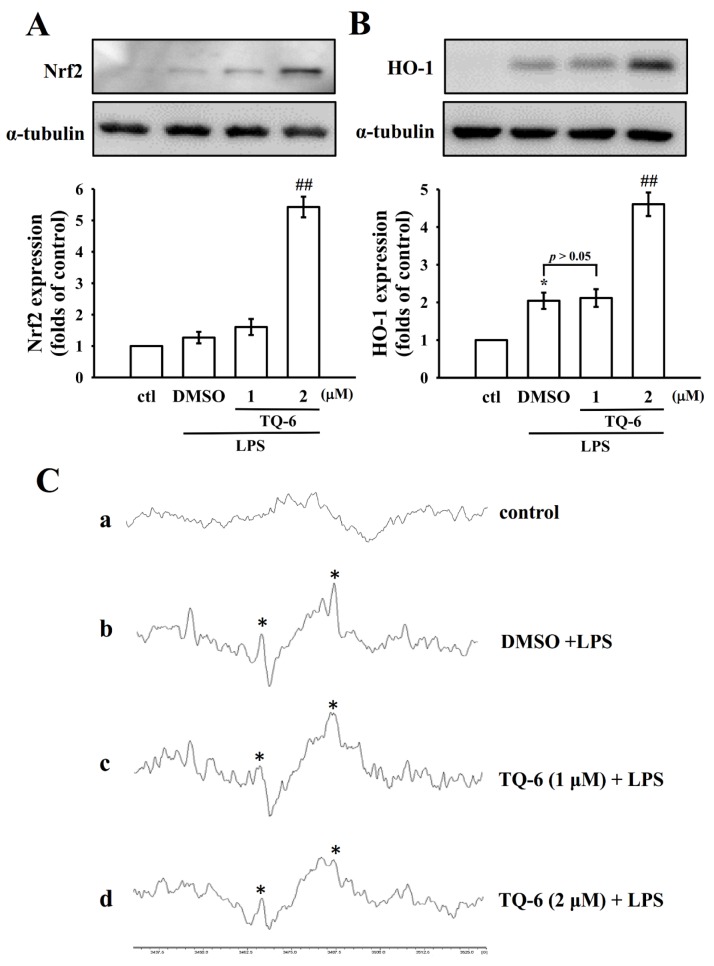
Effects of TQ-6 on Nrf2/HO-1 expression and OH^•^ free radical formation in LPS-stimulated microglia. Cells were treated with 0.1% DMSO or TQ-6 (1 or 2 μM) for 30 min followed by LPS (1 μg/mL) for 24 h. The expression of (**A**) Nrf2 and (**B**) HO-1 was evaluated using an immunoblotting assay. (**C**) For another study, the cells were treated with (**a**) a control (0.1% DMSO), (**b**) 0.1% DMSO, or TQ-6 at (**c**) 1 µM or (d) 2 µM, followed by LPS (1 µg/mL) to trigger OH^•^ formation. Profile C represents four independent experiments, and the asterisk (*) indicates OH^•^ formation. Data are presented as mean ± SEM (*n* = 4). * *p* < 0.05 compared with control (ctl); ^##^ *p* < 0.01 compared with the LPS-treated cells.

Nrf2 is involved in regulating inflammatory and antioxidative responses. Increased expression of Nrf2-dependent HO-1 plays a major role in the inhibition of oxidative stress-induced cell damage. Thus, this study examined the effects of TQ-6 on the Nrf2 and HO-1 expression in the microglia. The results indicated that LPS-treated cells exhibited a slightly enhanced expression of Nrf2 and HO-1 compared with the resting cells. However, TQ-6 pretreatment obviously increased LPS-induced Nrf2 and HO-1 expression (Figure 3A,B). Additionally, TQ-6 (1 or 2 μM) significantly reduced LPS-induced OH^•^ formation in microglia, as observed through ESR spectrometry (asterisk; Figure 3C).

### 3.4. TQ-6 Diminished MCAO-Induced Brain Infarct Volume and Edema in Mice

**Figure 4:**
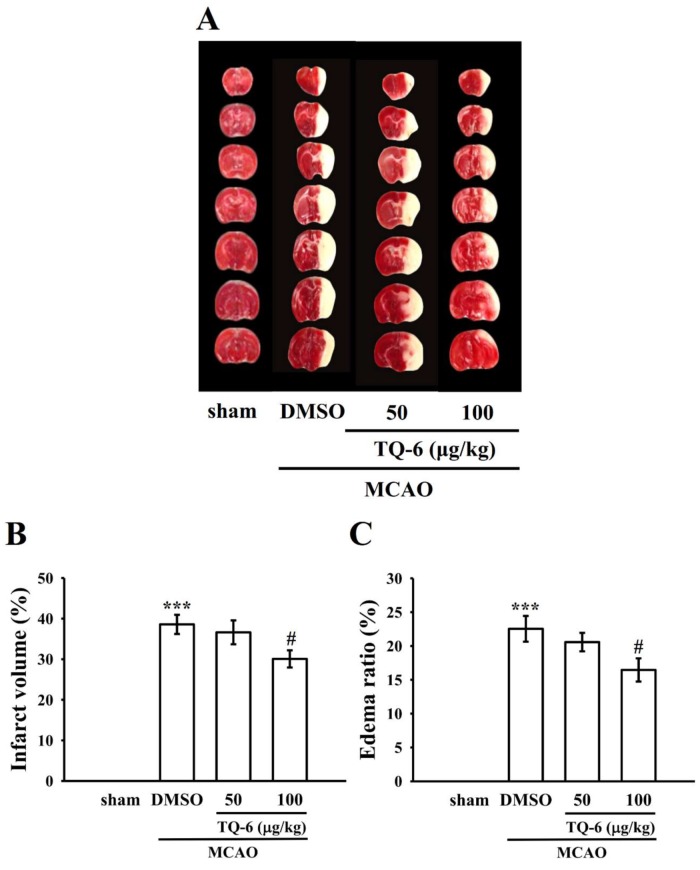
Protective effects of TQ-6 against cerebral ischemic stroke in mice. (**A**) Coronal sections of 2,3,5-triphenyltetrazolium chloride (TTC)-stained brains after middle cerebral artery occlusion (MCAO) in the sham operation group and the groups treated with either 0.1% DMSO or TQ-6 (50 and 100 μg/kg, intraperitoneal) for 30 min, followed by embolic occlusion, as described in the Materials and Methods section. Densitometric analysis for measuring the (**B**) infarct volume and (**C**) edema ratio after treatment with TQ-6 against embolic stroke in mice. Data are presented as mean ± SEM (*n* = 8). *** *p* < 0.001 compared with the sham operation group; ^#^ *p* < 0.05 compared with the MCAO group.

The neuroprotective effect of TQ-6 was estimated by assessing the brain infarct volumes and edema 24 h after ischemia. Figure 4A presents TTC-stained sections of mice in the sham operation, MCAO-treated, and TQ-6-treated groups. No infarction was evident in the sham operation group, whereas the development of extensive lesions was apparent on the striatum and lateral cortex in the MCAO group (Figure 4A). A significant (*p* < 0.05) reduction in the infarct volume was observed in the TQ-6-treated group (100 µg/kg) compared with that in the sham operation group (Figure 4A,B).

In addition, brain water content was measured to analyze MCAO-induced brain edema. Compared with the sham operation group, the ratio of brain edema was higher in the MCAO group (Figure 4C; *p* < 0.001 vs. sham). Compared with the MCAO group, the TQ-6-treated group (100 µg/kg) exhibited a considerable reduction in brain edema (Figure 4C).

### 3.5. TQ-6 Inhibited Aggregation and OH^•^ Formation in Mice Platelets

**Figure 5:**
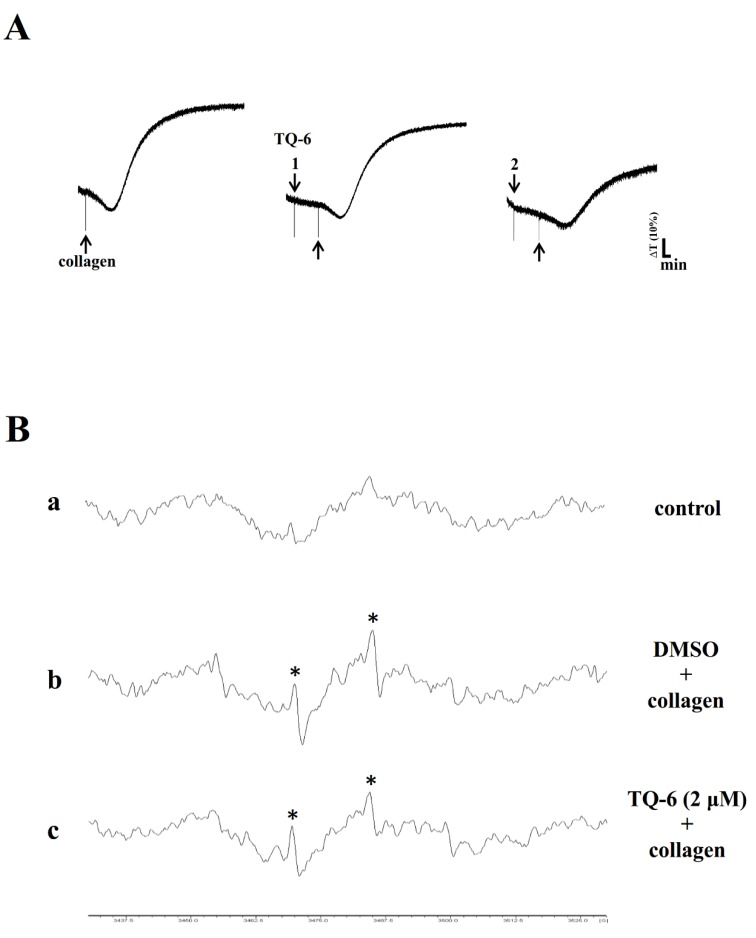
TQ-6-induced suppression of platelet aggregation and OH^•^ formation in collagen-activated mice platelets. (**A**) platelet-rich plasma (PRP) was preincubated with 0.1% DMSO or TQ-6 (1 or 2 μM), and subsequently treated with 2 μg/mL collagen to induce platelet aggregation. (**B**) For the ESR analysis, platelet suspensions were incubated with (**a**) only 0.1% DMSO (control) or preincubated with (**b**) 0.1% DMSO or (**c**) 2 μM TQ-6, and subsequently treated with 2 μg/mL collagen. The profiles of A and B represent four independent experiments, and the asterisk (*) indicates OH^•^ formation.

Platelets are involved in atherosclerotic plaque formation and are a cause of inflammatory reaction (i.e., free radicals), which may be a significant root of atherothrombosis [26]. Therefore, the effect of TQ-6 on platelet aggregation in PRP of mice was examined. TQ-6 (1–2 µM) substantially inhibited collagen-induced platelet aggregation in a concentration-dependent manner (Figure 5A). Moreover, compared with the resting control (Figure 5Ba), OH^•^ radical formation was noted during pretreatment with 0.1% DMSO, followed by the addition of collagen to platelet suspensions (Figure 5Bb). This was evidenced by the ESR signals (asterisk). TQ-6 (2 µM) significantly reduced OH^•^ formation in collagen-stimulated platelets (Figure 5C).

## 4. Discussion

Inflammatory events are particularly correlated with the progression of stroke and neurodegenerative diseases [27]. Inflammation of brain cells is induced through the activation of microglia, which secrete key inflammatory mediators and cause neuronal damage [28]. Microglia are principally involved in immune functions; moreover, when triggered, they exhibit macrophage-like functions, including the production of inflammatory mediators [29]. However, abnormal microglia activation is toxic for neurons because of the massive production of inflammatory and neurotoxic substances, such as proinflammatory cytokines, NO, and ROS [30]. Therefore, anti-inflammatory therapy-related intervention in microglia activation is critical for the treatment of numerous neurodegenerative conditions. This study investigated the effects of TQ-6 on LPS-induced inflammatory response in microglia, MCAO-induced ischemic stroke, and platelet aggregation in mice. The results revealed that TQ-6 inhibited LPS-induced inflammatory iNOS/COX-2 expression and NO production through the inhibition of NF-κB and activation of the Nrf2/HO-1 signaling pathway. Additionally, MCAO-induced brain infarction and edema, in vitro mice platelet aggregation, and OH^•^ free radical production were suppressed by TQ-6. Thus, TQ-6 enhances anti-neuroinflammatory effects and possesses therapeutic potential for inhibiting brain tissue damage in the presence of neuroinflammatory diseases.

Microglia react intensely to an LPS insult and produce NO and numerous inflammatory cytokines. NO is produced by iNOS and is defined as a toxic mediator in microglia-mediated brain inflammation [31]. Hence, substances or drugs that can obstruct iNOS expression are advantageous for treating neurological conditions with an overproduction of NO. COX-2 is crucial in neuroinflammation associated with brain diseases; treatment with selective COX-2 inhibitors diminishes brain inflammation and increases neuronal survival in ischemia and neurodegenerative diseases [32]. TQ-6 expressively inhibited NO production and iNOS/COX-2 expression in LPS-stimulated microglia. Thus, TQ-6 can diminish neuroinflammation caused by ischemic stroke through the regulation of microglia-mediated inflammatory molecule production.

The transcription factor NF-κB is an essential upstream regulator of inflammatory mediators, such as NO, and well known as the major target for treating inflammatory diseases [33]. Several lines of evidence have suggested that the recurrent stimulation of microglia can recruit and in turn activate NF-κB, leading to the expression of proinflammatory cytokines and inflammatory mediators [34]. Studies have indicated that drugs may mitigate the neuropathology of ischemic stroke through the inhibition of NF-κB signaling in microglia [35]. Nrf2 is the principal transcription factor that plays a vital role in regulating inflammatory and antioxidative responses. Generally, Nrf2 is impounded in the cytoplasm and combined with its inhibitor Keap1. Upon activation, Nrf2 translocates to the nucleus and controls the transcription of antioxidant proteins such as HO-1 [36]. Previous investigations have revealed that Nrf2 knockout mice are oversensitive to LPS-induced neuroinflammation [37]. Furthermore, Nrf2 activation might attenuate neuroinflammation through the modulation of NF-κB-mediated inflammatory mediators in the microglia [9]. In addition, compounds that inhibit NF-κB activation induce Nrf2 activation [38]. Thus, this study explored the effects of TQ-6 on the NF-κB and Nrf2 signaling pathways. The results revealed that TQ-6 inhibited the NF-κB expression and nuclear translocation, and increased the expression of Nrf2 and HO-1.

Ischemic stroke-mediated neurodegenerative diseases are vigorous processes induced by microglia. Stimulation of microglia is the initial step of the inflammatory process, and their activation is speculated to peak two to three days following ischemia, and persist for several weeks. The activation of microglia was amplified in the ipsilateral hemisphere of mice with stroke, but it remained at basal levels in the contralateral hemisphere [39]. In the ischemic region, microglia can phagocytose tissue debris as well as secrete proinflammatory cytokines, which cause additional destruction. Abnormal microglial activation expressively augmented the infarction volume after stroke [40], supporting the crucial role of microglia after ischemic stroke. Brain infarct size and edema volume are strongly correlated with neurological deficit. Honoor et al. [41] reported that the complexes of Cu(ii), Zn(ii), Mn(ii), and Ni(ii) demonstrated a dose-dependent reduction of edema volume in rats. In this study, MCAO triggered significant brain infarction and edema; however, TQ-6 repressed these deficits in a dose-dependent manner. Nrf2 plays an essential role in protecting brain cells from ischemic stroke injury, whereas Nrf2 gene depletion increases the cerebral infarction ratio and neurological deficits in ischemia reperfusion rats [42]. Nrf2 knockout exacerbates cerebral infarction and neural function defects in MCAO rats [42]. Therefore, the augmented effect of TQ-6 on Nrf2/HO-1 in the microglia might be a critical therapeutic target for stroke treatment.

The significance of arterial thrombosis includes the event of stroke, possibly signifying a vigorous process with thrombosis [43]. Platelets are critical for thrombus formation initiated by blood flow restriction, which indicates that platelets have a role in thromboembolic disease. The advantages of antiplatelet compounds or drugs are frequently suggested to patients with embolic stroke [43,44]. Cilostazol, clopidogrel, dipyridamole, and aspirin are commercially available antiplatelet drugs with serious undesirable side effects, such as headache, internal bleeding, prolonged bleeding time, and gastrointestinal bleeding [45]. Drugs that inhibit platelet aggregation, reducing myocardial infarction and ischemic stroke, and are associated with low bleeding rates [46]. This study noted that TQ-6 inhibited collagen-induced platelet aggregation. ROS play a destructive role in ischemia-induced neuronal injury. Ischemia-induced ROS may damage biomolecules in the brain, such as lipids, proteins, and nucleic acids, leading to brain dysfunction and cell death [47]. The results of this study revealed that OH^•^ was expressively augmented in collagen- and LPS-stimulated platelets and microglia, respectively. However, TQ-6 significantly reduced the elevated production of OH^•^ by both cells, indicating that the protective effects of TQ-6 against microglia activation, ischemic stroke, and platelet aggregation may partly be connected with OH^•^ suppression. One limitation of the studies presented in this paper is that control experiments with the separate TQ-6 and the ligands were not performed to assess whether or not the effects observed were not only a cumulative effect. Moreover, the absence of a docking analysis is considered to be another limitation of this study, as the molecular docking model is considered a significant tool for studying the relationships of ligands with receptors.

## 5. Conclusions

In this study, TQ-6 reduced NO production and iNOS/COX2 expression in microglia through its effect on the Nrf2/HO-1 and NF-κB signaling pathways. While attempting to enumerate brain damage reduction by TQ-6 using our MCAO model, we found that TQ-6 might regulate microglial function, suggesting its potential for treating neurodegenerative diseases such as cerebral ischemic stroke. In addition, the OH^•^ scavenging effects of TQ-6 may, at least partly, contribute to its neuroprotective efficacy in ischemic brain damage. Thus, this work recommends TQ-6 as a potential preventive or therapeutic drug for treating ischemic stroke-related diseases.
